# Distinct predictive biomarker candidates for response to anti-CTLA-4 and anti-PD-1 immunotherapy in melanoma patients

**DOI:** 10.1186/s40425-018-0328-8

**Published:** 2018-03-06

**Authors:** Priyanka B. Subrahmanyam, Zhiwan Dong, Daniel Gusenleitner, Anita Giobbie-Hurder, Mariano Severgnini, Jun Zhou, Michael Manos, Lauren M. Eastman, Holden T. Maecker, F. Stephen Hodi

**Affiliations:** 1 Institute for Immunity, Transplantation, and Infection, Stanford University School of Medicine, Stanford, CA USA; 2 Center for Immuno-oncology, Dana-Farber Cancer Institute and Harvard Medical School, Boston, MA USA; 3 Department of Biostatistics and Computational Biology, Dana-Farber Cancer Institute, Boston, MA USA; 4 Department of Medical Oncology, Dana-Farber Cancer Institute and Harvard Medical School, Boston, MA USA; 5 Melanoma Disease Center, Dana-Farber Cancer Institute and Harvard Medical School, Boston, MA USA; 6 Dana-Farber Cancer Institute, 450 Brookline Avenue, Boston, MA 02215 USA

## Abstract

**Background:**

While immune checkpoint blockade has greatly improved clinical outcomes in diseases such as melanoma, there remains a need for predictive biomarkers to determine who will likely benefit most from which therapy. To date, most biomarkers of response have been identified in the tumors themselves. Biomarkers that could be assessed from peripheral blood would be even more desirable, because of ease of access and reproducibility of sampling.

**Methods:**

We used mass cytometry (CyTOF) to comprehensively profile peripheral blood of melanoma patients, in order to find predictive biomarkers of response to anti-CTLA-4 or anti-PD-1 therapy. Using a panel of ~ 40 surface and intracellular markers, we performed in-depth phenotypic and functional immune profiling to identify potential predictive biomarker candidates.

**Results:**

Immune profiling of baseline peripheral blood samples using CyTOF revealed that anti-CTLA-4 and anti-PD-1 therapies have distinct sets of candidate biomarkers. The distribution of CD4^+^ and CD8^+^ memory/non-memory cells and other memory subsets was different between responders and non-responders to anti-CTLA-4 therapy. In anti-PD-1 (but not anti-CTLA-4) treated patients, we discovered differences in CD69 and MIP-1β expressing NK cells between responders and non-responders. Finally, multivariate analysis was used to develop a model for the prediction of response.

**Conclusions:**

Our results indicate that anti-CTLA-4 and anti-PD-1 have distinct predictive biomarker candidates. CD4^+^ and CD8^+^ memory T cell subsets play an important role in response to anti-CTLA-4, and are potential biomarker candidates. For anti-PD-1 therapy, NK cell subsets (but not memory T cell subsets) correlated with clinical response to therapy. These functionally active NK cell subsets likely play a critical role in the anti-tumor response triggered by anti-PD-1.

**Electronic supplementary material:**

The online version of this article (10.1186/s40425-018-0328-8) contains supplementary material, which is available to authorized users.

## Background

Immune checkpoint blockade with anti-CTLA-4 and anti-PD-1/PD-L1 has improved clinical responses and long term survival benefit for patients with advanced melanoma and other cancers [1–4]. Nevertheless, there persists a need for predictive biomarkers that facilitate patient selection and treatment decisions, since not all patients respond, and some patients respond better to one therapy versus another [5, 6].

Immune monitoring of peripheral blood is attractive for generating predictive biomarkers for cancer immunotherapy, due to the ease of accessing blood versus tumor tissue. Blood is also more homogeneous compared to tumors, making the sampling of blood easier and more consistent. However, the number of potential immune cell subsets and functions to monitor, are large. Most studies to date have used conventional flow cytometry, which limits the number of markers that can be simultaneously detected. To overcome this limitation, we used mass cytometry (CyTOF®) to extensively detect different cell lineages, activation markers, cytokines and cytotoxicity markers (Table 3). Unlike conventional flow cytometry which uses fluorophore-tagged antibodies, CyTOF uses metal ion tagged antibodies, allowing the combination of a larger number of antibodies for multiparametric analyses. Moreover, CyTOF is based on the principle of mass spectrometry for the detection of metal tags, avoiding the spectral overlap seen with flow cytometry [7].

In this study, we analyzed pre-treatment peripheral blood mononuclear cell (PBMC) samples from melanoma patients receiving anti-CTLA-4 or anti-PD-1, to investigate potential correlations in baseline immune features and clinical outcomes. Using a combination of various analytic approches, we identified distinct potential predictive biomarkers in PBMC for anti-CTLA-4 and anti-PD-1 treated patients.

## Methods

### Sample selection

In this retrospective study, 67 pre-treatment PBMC samples from patients treated with either anti-CTLA-4 or anti-PD-1 monotherapy were collected in accordance with an Institutional Review Board (IRB)-approved protocol. All patients provided consent prior to blood collection. Patients were treated with dosages and schedules that are FDA-approved for the treatment of metastatic melanoma. Baseline blood was drawn before the first administraton of anti-CTLA-4 or anti-PD-1. Patients treated with other therapeutics prior to or after the checkpoint blockade monotherapy were not excluded, provided baseline PBMCs were available. Patients who were treated with both anti-CTLA-4 and anti-PD-1 (at entirely different periods of time) were grouped into either the anti-CTLA-4 or anti-PD-1 cohort, based on the availability of baseline blood. Kaplan-Meier analysis showed that median overall survival was 46.7 months in the anti-CTLA-4 cohort, and was not yet reached (at 42 months) for the anti-PD-1 cohort (Additional file 1: Figure S1A and B). Median progression-free survival was 5 months in the anti-CTLA-4 group, and 7 months in the anti-PD-1 group (Additional file 1: Figure S1C and D).

Clinical responses such as complete response (CR), partial response (PR), stable disease (SD), and progressive disease (PD) (disease progression within 180 days) were determined based on Response Evaluation Criteria In Solid Tumors (RECIST) 1.1 [8]. Patients were further categorized as responders based on progression-free survival for at least 180 days, while non-responders were patients who had disease progression within 180 days (Table 1).

**Table 1:** Age and gender distribution of selected patients

		anti-CTLA-4		anti-PD-1	
		responders	non-responders	responders	non-responders
Age	< 30	0	1	0	0
	30–39	0	1	2	0
	40–49	1	4	2	2
	50–59	3	0	6	6
	60–69	4	5	4	6
	> 70	2	3	7	5
	Ave. age (years)	62.3	56.4	61.3	61.4
	STDEV (years)	10.9	17.7	15.3	11.6
	*p* value (U test)	0.35		0.81	
Gender	Female	4	4	8	7
	Male	6	10	13	12
Clinical response	CR	0		2	
	PR	2		12 (3 borderline CR)	
	SD	8		7	
	PD		14		19

Age and gender distribution of patients in the anti-CTLA-4 and anti-PD-1 cohorts in relation to clinical response are shown. More details regarding patient sample selection can be found in the Materials and Methods section. *p* values shown are from U test between responders and non-responders. *p* < 0.05 was considered statistically significant

Baseline PBMC samples from 40 patients treated with anti-PD-1 included 21 responders and 19 non-responders. Among these 21 patients, there were 2 CR patients, 12 PR patients, and 7 SD patients. Baseline PBMC samples from 27 patients treated with anti-CTLA-4 included 12 responders and 15 non-responders. Of these samples, 1 non-responder and 2 responder samples were excluded due to poor cell recovery (< 20,000 live intact cells collected). This resulted in 24 samples available for analysis in the anti-CTLA-4 cohort. Among the 10 responders, there were 2 PR patients, and 8 SD patients. No complete response patients were available. Among the 8 female patients treated with anti-CTLA-4, 4 were responders. Among the 16 male patients, 6 were responders. In the anti-PD-1 cohort, 8 out of 15 female patients, and 13 out of 25 male patients were responders. Initial assessment showed that age and gender did not affect the clinical response of patients from either therapy (Table 1). Among the 24 anti-CTLA-4 patients, 18 were treatment naïve. Among the remaining 6 treatment experienced patients, who were all non-responders, 3 had experienced chemotherapy, 1 had been given IFNγ therapy, and 2 had previously been treated with radiation therapy. Among the 40 anti-PD-1 patients, 14 were treatment naïve, 1 patient had experienced prior chemotherapy, 1 had been treated with NeoVax, and 1 with high dose IL-2. 23 patients had experienced prior checkpoint blockade therapy: among them, 3 had previously been given anti-PD-1 (nivolumab), and 20 had been treated with anti-CTLA-4 in various combinations (Table 2).

**Table 2:** Patient treatment history for anti-CTLA-4 and anti-PD-1 treated patients

Anti-CTLA-4 patients treatment history					
	Total patients	Treatment naïve	Treatment experienced	Checkpoint blockade experienced	
Responders	10	10	0	0	
Non-responders	14	8	6	0	
Total patients	24	18	6	0	
Anti-CTLA-4 patients treatment experienced					
	Treatment experienced		Ave. months between end of prior treatment and anti-CTLA-4 therapy	Range (months)	
Responders	0		N/A	N/A	
Non-responders	6		2.5	< 1~ 11	
Anti-PD-1 patients treatment history					
	Total patients	Treatment naive	Treatment experienced	Checkpoint blockade experienced	Nivolumab experienced
Responders	21	8	13	12	1
Non-responders	19	6	13	11	2
Total patients	40	14	26	23	3
Anti-PD-1 patients treatment experienced					
	Treatment experienced	Ave. months between end of prior treatment and anti-PD-1 therapy		STDEV (months)	U test p value between groups
Responders	13	6.3		7.1	0.59
Non-responders	13	4.5		6.6	

The above tables list the number of patients who were treatment naïve and treatment experienced in either the anti-CTLA-4 or anti-PD-1 cohort. In the anti-CTLA-4 cohort, 6 out of 24 patients were treatment experienced. None of them had experienced prior checkpoint blockade therapies. The average and range of time window (months) from end of prior therapy of any kind and start of anti-CTLA-4 are shown. Among anti-PD-1 patients, the majority of treatment experienced patients had prior checkpoint blockade therapies (23/26). Of these, 3 had prior nivolumab (in combination or sequential therapies with ipilimumab and other modalities). The average time window (months) from end of prior therapy of any kind and start of anti-PD-1 in both responders and non-responders are shown. Standard deviation of the time window in responders and non-responders are listed. Mann-Whitney U test was used to compare responders and non-responders

### PBMC collection and storage

Baseline pre-treatment blood was drawn according to the treatment protocol. The blood samples were subsequently processed at the Center for Immune-Oncology (CIO) of the Dana-Farber Cancer Institute. Peripheral blood mononuclear cells (PBMCs) were isolated from sodium heparin vacutainer blood samples by density gradient separation with Ficoll-Paque PLUS (GE Healthcare Biosciences, Uppsala, Sweden) within 6 h of collection. Blood was diluted 1:1 with phosphate buffered saline (PBS) and slowly layered on 12 ml of Ficoll-Paque PLUS in a 50 ml conical tube by pipetting carefully down the side of the tube with a transfer pipette. Tubes were centrifuged at 436 RCF for 20 min. PBMCs were aspirated from the density gradient using a transfer pipette. The collected cells were washed, and a small volume (10 μl) from each sample was used for cell counting on a Countess automated cell counter (Invitrogen, Carlsbad, CA). Live and dead cells were distinguished by trypan blue staining. Cells were then pelleted at 272 RCF for 5 min, re-suspended in Fetal Bovine Serum (FBS) (Life Technologies, Carlsbad, CA) + 15% dimethylsulfoxide (DMSO), and 4–6 × 10^6^ cell aliquots were prepared. Cells were frozen overnight at -80 °C @ -1 °C/min and transferred to liquid nitrogen for cryogenic storage until assay.

### Sample preparation and staining for CyTOF

Patient samples were processed for intracellular cytokine staining by CyTOF as previously described [9, 10]. Briefly, frozen PBMC were thawed and washed twice in complete medium (RPMI supplemented with Pen-Strep and L-glutamine). All samples from the anti-CTLA-4 as well as the anti-PD-1 cohorts, upon thawing, had viability in the range of 89–98%, and most yielded 3-6 × 10^6^ viable cells. A few samples were low at 1.5 × 10^6^, and some samples gave more than 100% recovery, suggesting that there was some variability in the number of cells that were frozen. All cell counts were obtained using a Vi-Cell XR cell viability analyzer (Beckman Coulter, Brea, California). After thawing, samples were split into 2 experimental conditions: unstimulated and PMA + Ionomycin, with 2 × 10^6^ cells (or maximum available) per condition. They were rested in 96-well U-bottom plates overnight at 37 °C, 5% CO_2_. After resting, secretion inhibitors brefeldin A (5 μg/ml) and monensin (5 μg/ml) (Sigma-Aldrich, St. Louis, MO) were added to both unstimulated and PMA + Ionomycin conditions. Anti-CD107a antibody conjugated to 151Eu (Fluidigm, South San Francisco, CA) was also added to both conditions, concentration as recommended by the supplier. Additionally, for PMA + Ionomycin stimulation, we added 10 ng/ml PMA and 1 μg/ml Ionomycin, while the unstimulated samples were treated with only secretion inhibitors along with anti-CD107a-151Eu antibody. All samples were incubated for 4 h at 37 °C. Thereafter, 2 mM EDTA was added for 15 min at room temperature. Cells were washed twice in CyFACS buffer (1X diluted from 10X PBS, (Rockland Immunochemicals, Pottstown, PA) supplemented with 0.1% BSA and 0.05% sodium azide). A cell-surface antibody cocktail consisting of pre-conjugated antibodies (Fluidigm, South San Francisco, CA) as well as in-house conjugated antibodies (Table 3) was prepared in CyFACS and added to the cells for 45 min on ice. All antibody cocktails were filtered using 0.1 μm spin filters (Millipore, Darmstadt, Germany) to remove possible antibody aggregates before staining. Cells were washed twice in CyFACS after surface staining, and 1:3000 ^115^In -DOTA Maleimide (5 mg/ml) live/dead stain was added for 30 min on ice. The cells were then fixed in 2% paraformaldehyde (PFA) in PBS at 4 °C overnight. These fixed cells were washed twice in 1X permeabilization buffer (eBioscience, San Diego, CA). Intracellular staining cocktail, also consisting of Fluidigm pre-conjugated as well as in-house conjugated antibodies was prepared in 1X permeabilization buffer and added to the cells for 45 min on ice (Table 3). After washing three times in CyFACS, cells were stained with Intercalator-Ir (Fluidigm) diluted as per the manufacturer’s recommendations in 2% PFA in PBS, and incubated for 20 min at room temperature. Finally, cells were washed twice in CyFACS and three times in MilliQ water. EQ Four Element Calibration Beads from Fluidigm were added as per the manufacturer’s directions prior to running. Data were acquired on a Helios mass cytometer (Fluidigm) in batches over two major time frames. Principal component analysis showed no significant difference between them (data not shown).

**Table 3:** CyTOF panel for the phenotypic and functional analysis of immune cell subsets

	Metal label	Specificity	Antibody clone
1	115In	*Dead cells*	n/a
2	140Ce	*Beads*	n/a
3	141Pr	CD25	M-A251
4	142Nd	CD19	HIB19
5	143Nd	IL-10	JES3-9D7
6	144Nd	IL-4	MP4-25D2
7	145Nd	CD4	RPA-T4
8	146Nd	CD8	RPA-T8
9	147Sm	CD20	2H7
10	148Nd	CD57	HCD57
11	149Sm	CTLA-4	14D3
12	150Nd	MIP-1β	D21–1351
13	151Eu	CD107a	H4A3
14	152Sm	TNFa	Mab11
15	153Eu	CD45RA	HI100
16	154Sm	CD3	UCHT1
17	155Gd	CD28	L293
18	156Gd	CD38	HB-7
19	157Gd	HLA-DR	G46–6
20	158Gd	CD33	WM53
21	159 Tb	GMCSF	BVD2-21C11
22	160Gd	CD14	M5E2
23	161Dy	IFNγ	4S.B3
24	162Dy	CD69	FN50
25	163Dy	TCRγδ	B1
26	164Dy	IL-17	N49–853
27	165Ho	CD127	A019D5
28	166Er	IL-2	MQ1-17 h12
29	167Er	CD27	L128
30	168Er	CD154 (CD40L)	24–31
31	169Tm	CCR7	150503
32	170Er	PD1	EH12.1
33	171Yb	Granzyme B	GB11
34	172Yb	PD-L2	24F.10C12
35	173Yb	Perforin	B-D48
36	174Yb	CD16	3G8
37	175Lu	PD-L1	29E.2A3
38	176Yb	CD56	NCAM16.2
39	191Ir	*DNA1*	n/a
40	193Ir	*DNA2*	n/a

A 40-marker CyTOF panel for the phenotypic and functional analysis of immune cell subsets in melanoma patients treated with anti-CTLA-4 or anti-PD-1 therapy is shown. The element and isotope of the metal tag conjugated to each antibody and non-protein subject (*italic*) is indicated under the ‘Metal Label’ column. ‘Specificity’ indicates the target recognized by the metal-conjugated antibody or non-protein subject. 193Ir/195Ir DNA Intercalator and 115In Maleimide DOTA live/dead stain facilitate the identification of live intact singlets while calibration beads (140Ce) are important for data pre-processing. Antibody clones are listed when applicable

### CyTOF data pre-processing and subsequent analysis

**Fig. 1:**
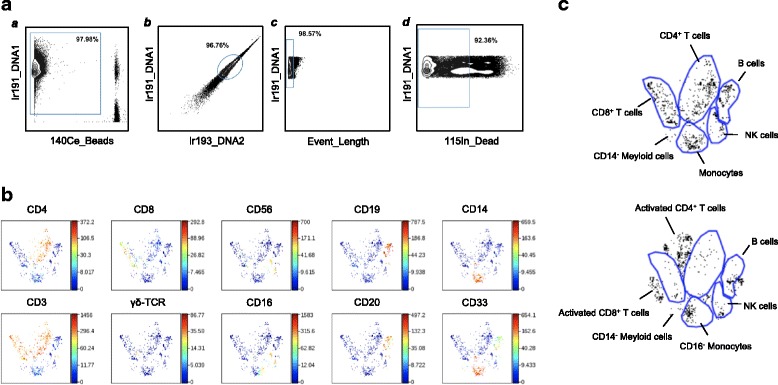
viSNE analysis of CyTOF data results in well-defined immune subset maps in PBMC. Preliminary gating of CyTOF data to define live intact single cells, was performed in CytoBank. Then viSNE mapping of the healthy donor PBMC and patient baseline PBMC are shown. **a** All ungated events were sequentially gated in Cytobank to identify live intact single cell events as described in Methods. *a)* 191Ir DNA1 and 140Ce beads were used to identify cells. *b)* Singlets were identified by gating on cells positive for the DNA markers 191Ir DNA1 and 193Ir DNA2. *c)* Event Length of singlets from *b)* was used to obtain intact singlets. *d)* Live intact singlets were obtained by gating for intact singlets from *c)* which were negative for dead cell marker 115In maleimide DOTA. Subsequent viSNE analysis was performed using live intact single cell events obtained from *d)*. **b** viSNE map of healthy donor PBMC showing distinct immune subsets. The heat spectrum associated with each graph indicates the relative expression level of each marker. **c** Various immune subsets were grouped into distinct islands on the viSNE map

Data were obtained in the form of .fcs files from the Helios instrument. The addition of EQ Four Element Beads allowed us to use the MATLAB-based normalization technique using bead intensities as described [11]. We used the Nolan Lab MATLAB normalizer available freely on github.com (https://github.com/nolanlab/bead-normalization/releases). Following this pre-processing, .fcs files were further analyzed in FlowJo version 10.1 for Mac (FlowJo LLC, Ashland, OR), or uploaded to Cytobank (www.cytobank.org) for subsequent viSNE and Citrus analysis (details below). Before viSNE and Citrus analysis, sequential gating was performed on Cytobank. ^191^Ir and ^193^Ir DNA intercalator and ^140^Ce beads, as well as the event length parameter were used to discern intact singlets from debris and cell aggregates. ^115^In-DOTA Maleimide live/dead staining was then used to identify live intact singlets (Fig. 1a). A similar initial gating strategy to detect live intact singlets was used while analyzing CyTOF data by FlowJo (Additional file 1: Figure S2A). All other cell populations were identified as indicated in the respective figures (Additional file 1: Figure S2B-I). Data shown in this study are in the form of relative frequencies as derived from FlowJo gating. All results presented in this study are from unstimulated samples, except when PMA + Ionomycin stimulation is explicitly stated. GraphPad Prism 6.0d (GraphPad Software, Inc., La Jolla, CA) was used for graphing and statistics. The non-parametric Mann-Whitney U test was used, and *p* values less than 0.05 were considered significant (*p < 0.05, **p < 0.01). Frequencies of cell subsets/expression markers from FlowJo gating were used for multivariate analysis (described below).

### viSNE and citrus analysis

viSNE is an automated dimensionality reduction algorithm available in CytoBank [12]. The normalized CyTOF .fcs files of all patient baseline PBMC, and one healthy donor PBMC, including both the unstimulated and PMA + Ionomycin stimulated samples were uploaded to CytoBank. Sequential gating was performed on each file to separate live intact single cells from beads, aggregates, and debris as described above (Fig. 1a). Subsequent analyses were performed on live intact singlets. For viSNE analysis, patient .fcs files were categorized into responders and non-responders for either anti-CTLA-4 or anti-PD-1 groups. 29 markers: CD25, CD3, CD4, CD8, CD19, CD20, CD56, CD14, TCRγδ, CD16, CD33, CD45RA, HLA-DR, CD27, CD28, CD38, CD57, CD69, CCR7, CD127, CD154, CTLA-4, PD-1, PD-L1, PD-L2, GranzymeB, CD107a, GM-CSF, MIP-1β were used to build the map. Cytokines with low expression were excluded because they would not contribute to the structure of the map. A total of 99,904 events were extracted by the algorithm using equal sampling of 892 events per .fcs file. Once the maps were made, the expression level and distribution of each marker of interest could be visualized by coloring based on each one.

Citrus (cluster identification, characterization, and regression) is an automated algorithm designed to discover statistically significant stratifying features across known endpoints [13]. In the Citrus analysis, anti-CTLA-4 and anti-PD-1 files were assessed separately. Files were grouped into responders and non-responders. Seven surface lineage markers, CD19, CD4, CD8, CD3, CD33, CD14, and CD56 were used as clustering channels. Unlike SPADE and some other clustering algorithms, Citrus presents events redundantly in the tree, with each parent cluster containing all the events in its children, and children of children. This starts with the center cluster, which contains all events in the analysis. Each parent cluster has no more than two direct children. This clustering process continues until it reaches the user-defined cluster size limit (set as a percentage of all events analyzed; we used 5%). A parent cluster may have only one direct child cluster if the second cluster is below the size limit. Each cluster has an identifying number associated with it. Median signal intensity and cluster abundance are two modes in Citrus, which are mutually exclusive. In our analysis, we focused on the signal intensity mode, but also ran in cluster abundance mode. The median signal intensities of 29 out of 46 CyTOF channels were analyzed, with all seven lineage markers used for clustering excluded by default in the algorithm, and all non-protein channels: Time, event_length, 115In_Dead, 140Ce_Beads, 191IrDNA1, 193IrDNA2, center, offset, width, residual excluded by the investigator. Nearest Shrunken Centroid (PAMR) model was selected as the predictive algorithm.

### Multivariate analysis

The 210 cell population fractions generated in FlowJo were ranked using a Wilcoxon Ranksum test according to response. Specifically, we used the wilcox.test function of the stats package in R to generate raw *p*-values, which were corrected for the false discovery rate using the Benjamini-Hochberg procedure [14]. In addition, the fractions generated by FlowJo were used as independent features to generate a biomarker based on an elastic net classifier [15]. Since no independent dataset was available, a leave-one-out cross-validation (LOOCV) strategy was used to ensure an unbiased estimation of classification performance. Within each partition of the LOOCV one sample was left out for testing, feature selection based on a Wilcoxon test was performed to get a ranking of feature importance and the top features were used to build an elastic net prediction model, which was used to predict the left out sample. This strategy is repeated until each sample has been left out once. LOOCV allows for the determination of the optimal number of features, and an estimation for the alpha and lambda parameters of the elastic net model. For each model, both the area under the receiver operating characteristic curve (AUC) and accuracy for the model that has a cutpoint equally weighing false positive and false negative predictions are reported. The optimal models were selected by maximizing AUC.

**Fig. 2:**
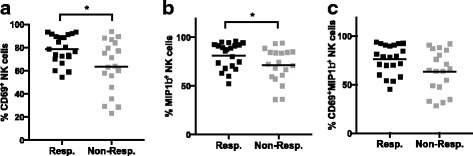
Non-responders to anti-PD-1 had lower CD69 and MIP-1β expressing NK cells following PMA + Ionomycin stimulation. Pre-treatment PBMC from melanoma patients who were responders or non-responders to anti-PD-1 therapy were stimulated with PMA + Ionomycin ex vivo, and analyzed by CyTOF. Frequencies of (**a**) CD69^+^, (**b**) MIP-1β^+^ and (**c**) CD69^+^MIP-1β^+^ NK cells are shown. *p < 0.05

For the anti-CTLA-4-treated group, the optimal parameters were 4 features (Fig. 2), alpha: 0.0 and lambda: 0.001. For the models build on the anti-PD-1 treated samples the optimal parameters were 25 features (data not shown), alpha: 0.9 and lambda 0.002. Both random forest [16] and shrunken centroid [17] classifiers were compared to the elastic net with inferior classification performance in both datasets. All analyses were performed using the R and the glmnet, pamr, randomforest and pROC packages.

## Results

### viSNE analysis of CyTOF data produces well-defined immune subset maps in PBMCs

The dimentionality reduction tool viSNE [12] was employed to turn high-dimensional CyTOF data from each PBMC sample into a two-dimensional map of immune cell subsets, with each dot in the map representing one event (cell). Live intact single cells gated from the unstimulated PBMC of a healthy donor (Fig. 1a) could be clearly grouped into distinct subsets (Fig. 1b), including B cells (CD19^+^CD20^+^), CD4^+^ T cells (CD3^+^CD4^+^), CD8^+^ T cells (CD3^+^CD8^+^), Natural Killer (NK) cells (CD3^−^CD56^+^CD16^+/lo^), and monocytes (CD33^+^CD14^+^). Natural Killer T (NKT) cells, (CD3^+^CD56^+^) as well as γδ T cells (CD3^+^TCRγδ^+^) also form distinct T cell populations on the viSNE map. (Fig. 1b). After PMA + Ionomycin stimulation, there was a clear shift in the location of various populations on the viSNE map (Fig. 1c). These differences in viSNE patterns of major immune cell clusters indicates that, in spite of being the same cell type, viSNE can detect differences in their expression patterns, and assigns them somewhat altered locations in 2-dimensional space. After stimulation, activated T cells are clustered together, but appear outside of the original CD4^+^ or CD8^+^ bubbles as seen in the unstimulated viSNE map (Fig. 1c, top and bottom panels).

viSNE maps were used to compare responders and non-responders to anti-CTLA-4 and anti-PD-1 therapies. The viSNE maps of patient baseline PBMCs shared the same basic structure as the healthy donor (data not shown). Closer examination revealed that the viSNE maps of some patients were not well-structured, with missing or significantly shrunken clusters in various subsets. Stronger CD45RA intensity was observed in the CD4^+^ and CD8^+^ compartments of non-responders to anti-CTLA-4 therapy (Additional file 1: Figure S3). No difference in CD45RA intensity was found in patients treated by anti-PD-1 (data not shown). viSNE served to visualize such differences well. However, it has limited ability to provide quantifiable results, and we therefore used manual analysis to further investigate the differences between responders and non-responders.

### Differential distribution of CD4^+^ and CD8^+^ T cell memory subsets in anti-CTLA-4 responders versus non-responders

The frequency of all major immune subsets was assessed using conventional supervised gating on FlowJo (Fig. 1a and Additional file 1: Figure S2B-I). In contrast to previous reports [18, 19], this analysis did not reveal obvious differences in lymphocyte and monocyte frequencies between responders and non-responders to anti-CTLA-4 or anti-PD-1 therapy (Additional file 1: Figure S2B and C). The frequencies of the other immune subsets, including B cells, T cells, NK cells, and NKT cells, as well as the CD8^+^ to CD4^+^ ratio, did not show significant differences between responders and non-responders to either therapy (Additional file 1: Figure S2 D-I). There was also no difference in γδ T cell frequencies between responders and non-responders. However, clone B1 was used for TCRγδ staining, which could potentially have been affected by simultaneous CD3 staining. Further investigation will be needed to evaluate the potential of the γδ T cell population as a biomarker.

**Fig. 3:**
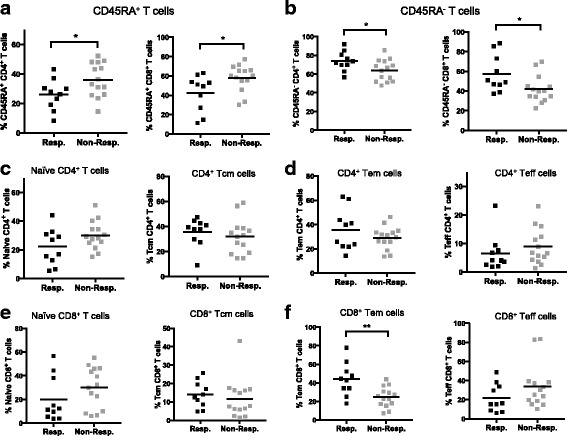
Melanoma patients who responded to anti-CTLA-4 therapy had higher frequencies of memory T cells in baseline PBMC. CyTOF data from baseline PBMC of melanoma patients treated with anti-CTLA-4. Frequencies of (**a**) CD45RA^+^ cells in CD4^+^ and CD8^+^ T cell compartments; (**b**) CD45RA^−^ cells in CD4^+^ and CD8^+^ T cell compartments. **c-f** Memory subsets of CD4^+^ and CD8^+^ T cells in responders versus non-responders. **c** Frequencies of naïve (CD45RA^+^CCR7^+^) and Central Memory (Tcm, CD45RA^−^CCR7^+^) CD4^+^ T cells. **d** Frequencies of Effector Memory (Tem, CD45RA^−^CCR7^−^) and Terminal Effector (Teff, CD45RA^+^CCR7^−^) CD4^+^ T cells. **c** Frequencies of naïve (CD45RA^+^CCR7^+^) and Central Memory (Tcm, CD45RA^−^CCR7^+^) CD8^+^ T cells. **d** Frequencies of Effector Memory (Tem, CD45RA^−^CCR7^−^) and Terminal Effector (Teff, CD45RA^+^CCR7^−^) CD8^+^ T cells. *p < 0.05; **p < 0.01

Since anti-CTLA-4 [20, 21] and anti-PD-1 [22, 23] target T cell checkpoint signaling pathways, we focused our attention on CD4^+^ and CD8^+^ T cells. We found that in anti-CTLA-4 treated patients, responders had significantly lower frequencies of CD45RA^+^ cells in both the CD4^+^ (*p* = 0.038) and CD8^+^ (*p* = 0.019) T cell compartments (Fig. 3a). The converse was also true for CD45RA^−^ cells (Fig. 3b). In anti-PD-1 treated patients, there were no apparent differencs in CD45RA expression between responders and non-responders (Additional file 1: Figure S4A and B). All samples were obtained prior to the initiation of immunotherapy, indicating that baseline CD45RA expression level in T cells correlates with clinical response to anti-CTLA-4 but not anti-PD-1 treatment.

CD45RA expression, in conjunction with CCR7, can be used to identify naïve (CD45RA^+^CCR7^+^), central memory (Tcm, CD45RA^−^CCR7^+^), effector memory (Tem, CD45RA^−^CCR7^−^) and terminal effector (Teff, CD45RA^+^CCR7^−^) cells [24]. In the anti-CTLA-4 treated patients, we found no significant differences in CD4^+^ T cell memory subsets between responders and non-responders (Fig. 3c and d). However, naïve T cells and Teff appeared lower in responders while Tcm and Tem tended to be lower in non-responders, although these trends did not reach statistical significance (Fig. 3c and d). Similar results were obtained using CD27 in place of CCR7 to define [25, 26] these memory T cell subsets (data not shown).

For CD8^+^ T cells, naïve and Teff tended to be lower in responders, although not statistically significant (Fig. 3e and f). Conversely, the frequencies of Tcm and Tem cells were higher in responders compared to non-responders to anti-CTLA-4 therapy (Fig. 3e and f). Among these immune subsets the CD8^+^ Tem frequency was significantly higher in responders to anti-CTLA-4 (*p* = 0.008) (Fig. 3f). In anti-PD-1 treated patients, there were no differences in CD4^+^ or CD8^+^ memory T cell subsets (Additional file 1: Figure S4 C-F). Since CD45RA^−^ T cells include central and effector memory T cells, our results show that baseline memory CD4^+^ and CD8^+^ T cells, and CD8^+^ effector memory T cells may be predictive biomarkers of response to anti-CTLA-4 treatment.

### MIP-1β and CD69 expressing NK cells are higher in patients who respond to anti-PD-1 therapy

**Fig. 4:**
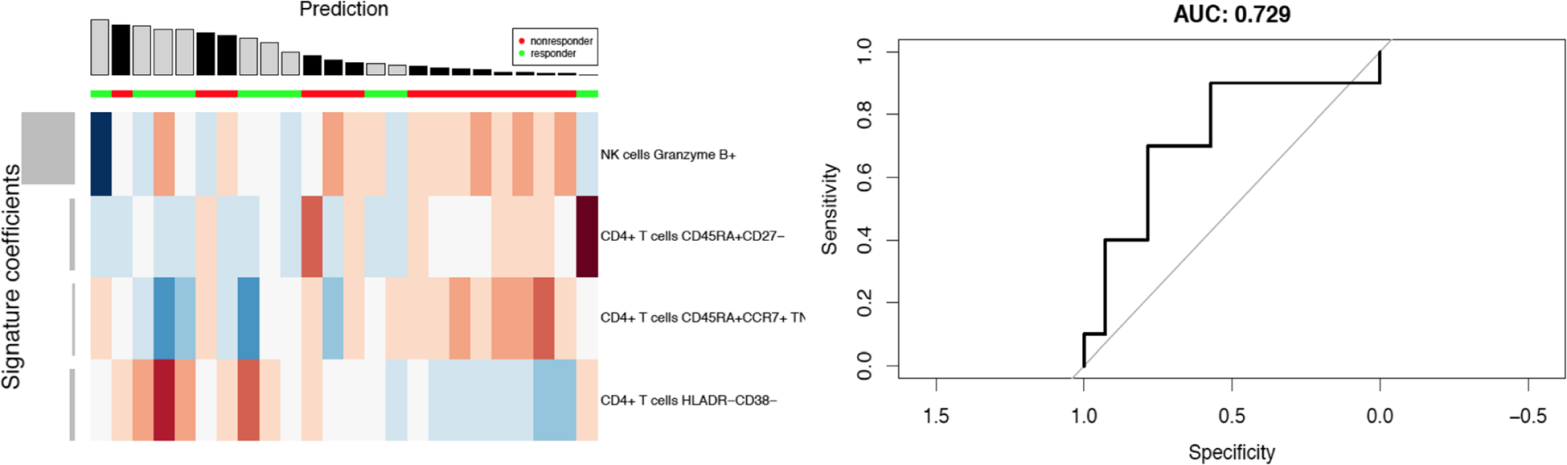
Multivariate predictive models for response to anti-CTLA-4 treatment. Multivariate analysis based on the FlowJo gating of CyTOF data was performed to generate a model that predicts clinical response to anti-CTLA-4 monotherapy. The heatmap is row-normalized and shows the four features used by the model with the highest area under the curve (AUC). Each value in the heatmap corresponds to a cell population as determined by manual gating, where red indicates a larger population and blue a smaller one. The signature coefficients (grey bars) on the left side indicate the elastic net weights, which corresponds to the importance in the prediction model. The clinical response of each patient is indicated in green (responder) and red (non-responder) as a horizontal bar above the heat map. The predictions of clinical response for each patient are indicated in grey (responders) and black (non-responders), where the height of the vertical bar represents the probability output of the predictor. The receiver operating characteristic (ROC) curve for the model is shown on the right

We further investigated the potential role of NK cell populations in predicting outcome to anti-CTLA-4 and anti-PD-1 therapy. In general, we gated NK cells as CD56^+^CD16^+^ from CD3^−^ lymphocytes. However, following PMA + Ionomycin stimulation, CD16 is downregulated, and so we modified this gate to detect CD56^+^CD16^lo^ NK cells, whose frequency was within 10% of the CD56^+^CD16^+^ NK cell population in the corresponding unstimulated sample. After analyzing the expression of functional markers on NK cells, we found that responders to anti-PD-1 therapy had significantly higher CD69^+^ NK cells (Fig. 4a) than non-responders, in PMA + Ionomycin stimulated PBMC. Also, the expression of MIP-1β in NK cells after stimulation was higher in responders compared to non-responders (Fig. 4b). There was no correlation between the expression of MIP-1β and CD69, indicating that these are independent predictors (data not shown). In order to investigate if combining MIP-1β and CD69 could provide greater predictive value, we gated CD69 and MIP-1β double positive NK cells, which formed a distinct CD69^+^MIP-1β^+^ population (Additional file 1: Figure S5). This CD69^+^MIP-1β^+^ NK cell population in stimulated samples seemed higher in anti-PD-1 responders as compared to non-responders, but did not reach statistical significance (*p* = 0.0538) (Fig. 4c). In unstimulated samples, we did not observe statistically significant differences between CD69^+^, MIP-1β^+^, or CD69^+^MIP-1β^+^ NK cells between responders and non-responders (data not shown). Interestingly, PMA + Ionomycin stimulated samples from the anti-CTLA-4 cohort did not show any differences in CD69^+^, MIP-1β^+^ or CD69^+^MIP-1β^+^ NK cells between responders and non-responders (Additional file 1: Figure S6). This suggests that CD69^+^ and MIP-1β^+^ NK cells are potential biomarker candidates specific to anti-PD-1 (but not anti-CTLA-4) immunotherapy.

In this study, our examination of Tregs (defined as CD4^+^CD25^+^CD127^lo^) did not show any significant correlation with response to anti-CTLA-4 or anti-PD-1 therapy (Additional file 1: Figure S7). We also did not find any association between CTLA-4 and PD-1 expression on either CD4^+^ or CD8^+^ T cells with clinical benefit (Additional file 1: Figure S8). The expression of PD-L1 and PD-L2 on all major immune subsets, including monocytes and NK cells, also did not correlate with clinical response (Additional file 1: Figure S9). We observed some variation in PD-L1 on monocytes (Additional file 1: Figure S9A) and PD-L2 on NK cells, perhaps due to variability in antibody lots (Additional file 1: Figure S9D). Both PD-L1 and PD-L2 were pre-conjugated antibodies purchased from Fluidigm (South San Francisco, CA). Importantly, within each cohort, the expression levels were comparable, and we did not observe any differences between responders and non-responders. The expression of intracellular cytokines such as IL-2, IL-4, Il-10, IFNγ, GM-CSF, etc. was assessed, and not found to be predictive of response to anti-CTLA-4 or anti-PD-1 therapy (data not shown). The only feature that was associated with clinical outcome was Granzyme B in NK cells, which was significantly higher in non-responders to anti-PD-1 (*p* = 0.0054) (Additional file 1: Figure S10A). In spite of the statistical significance, we observed that the expression levels were very high, and the actual percentages in responders and non-responders were close. We believe that further validation is needed to be able to accurately and conclusively interpret these data. Granzyme B expression in CD8^+^ T cells did not differ between responders and non-responders (Additional file 1: Figure S10B).

### Multivariate analysis helped to identify the optimal combination of multiple features that best correlate clinical response to either anti-CTLA-4 or anti-PD-1 therapy

Some strong correlations between clinical response and baseline PBMC markers were identified through immune phenotyping with our CyTOF panel (Table 3). However, none of the parameters measured could distinctly separate responders from non-responders, due to significant overlap of the parameter values in both groups. In further attempts to resolve this, a multivariate approach was adopted to investigate if a combination of parameters could distinguish responders and non-responders to anti-CTLA-4/anti-PD-1 treatment with greater sensitivity and specificity.

The multivariate analysis included all parameters generated by manual analysis. The frequency of each phenotypic and functional immune cell subset, including the expression level of every surface and intracellular protein, were assessed as independent features in a multivariate test. In order to reduce redundant information such as the reciprocity between CD45RA^+^ and CD45RA^−^, only one feature instead of both were used in the analysis. Using the Wilcoxon Ranksum Test, all cell subsets were ranked based on their feature importance with adjusted *p* values. Next, a leave-one-out cross-validation (LOOCV) strategy was adopted to ensure unbiased classification with an elastic net classifier (as described in Materials and Methods). As shown in Fig. 4, LOOCV allowed the determination of an optimal number of features for an elastic net model of prediction.

**Fig. 5:**
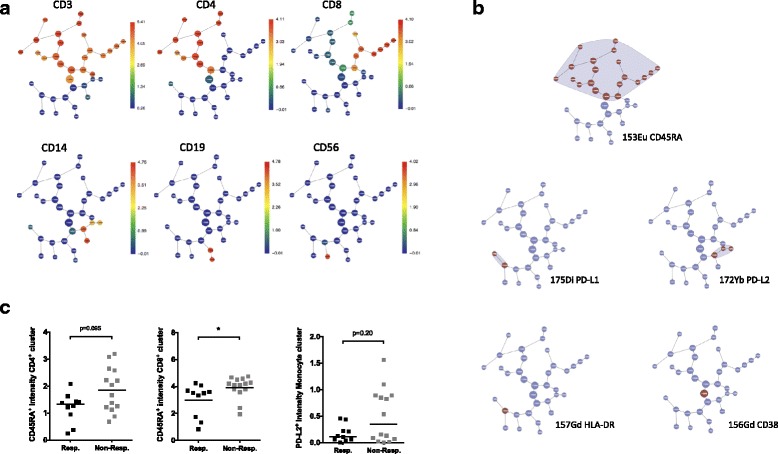
Parameters that correlate with anti-CTLA-4 and anti-PD-1 clinical response were verified by the automated Citrus algorithm. CyTOF .fcs files were analyzed using the Citrus algorithm to establish a predictive model for anti-CTLA-4 treatment. (**a**) Citrus clustering of immune subsets (represented by metal_marker channels) based on CyTOF data from baseline PBMCs of anti-CTLA-4 treated patients are shown as graphs. The distribution of each immune subset is presented as an individual graph. The heat spectrum associated with each graph indicates the expression level of each channel in a cluster. (**b**) A Nearest Shrunken Centroid (PAMR) model described the minimum number of channels and clusters needed to distinguish responders from non-responders to anti-CTLA-4 with the lowest error rate. (**c**) Comparisons of metal signal intensities (mean) of indicated channels are shown from left to right: CD45RA (153Eu) in base CD4^+^ and CD8^+^ clusters, and PD-L2 (172Yb) intensity in the base monocyte cluster in responders vs. non-responders to anti-CTLA-4 therapy. Two clusters from the Citrus predictive model in (**b**) are not demonstrated in (**c**) The cluster highlighted for the signal of HLA-DR (157Gd) is not associated with any major immune subsets in this analysis. The cluster highlighted for the signal of CD38 (156Gd) is the base cluster of total PBMC^§. §^CD38 expression on total PBMC was also assessed, and no significant difference between responders and non-responders was found in the univariate analysis.

In the anti-CTLA-4 treated group, a combination of four features were determined as optimal to predict clinical response, with an AUC value of 0.729 (Fig. 5). The features highlighted by the model include the frequencies of Granzyme B^+^ NK cells, CD4^+^ Teff cells (CD4^+^CD45RA^+^CD27^−^), CD45RA^+^CCR7^+^ TNFα^+^ CD4^+^ T cells and HLADR^−^CD38^−^ CD4^+^ T cells. These results were somewhat in agreement with the univariate analysis which highlighted memory subsets: CD45RA^+^ and CD45RA^−^ CD4^+^ and CD8^+^ T cells, as well as CD8^+^ Tem cells as potential predictive markers. As per the univariate analysis, the Granzyme B expression in NK cells tended to be higher in non-responders (*p* = 0.0054) but was not significant after correction for multiple testing (FDR = 0) (Additional file 1: Figure S10A).

A similar model was derived in the anti-PD-1 treated group, however the optimal model based on twenty-five features only achieves an AUC of 0.569 (data not shown), indicating little prediction value.

### Correlative features identified in FlowJo and multivariate analysis can be further validated by the citrus algorithm

The unsupervised algorithm Citrus was used to further validate the findings of the individual statistical tests and the multivariate modeling [13]. In the ani-CTLA-4 study, a predictive model was produced (Fig. 5a-c) that included the signal intensities of CD45RA in multiple clusters in CD4^+^ and CD8^+^ T cell branches, as well as CD38 expression in one of the CD4^+^ clusters and the base cluster, which included all PBMC events (Fig. 5a and b). The model also highlighted HLA-DR and PD-L1 expression on an uncharacterized cluster (Fig. 5b), but this cluster appears very heterogeneous when we analyzed the exported events by manual gating. PD-L2 expressing CD14^+^ monocytes were selected by the automated model too, although univariate analysis revealed that the difference between responders and non-responders were not significant. The identification of CD45RA expression as the major feature correlating with clinical response was consistent with the observations from univariate and multivariate analyses. However, Citrus was unable to precisely assess functional subsets that are defined by the expression of a combination of parameters, such as memory T-cell subsets or regulatory T cells (Tregs). There was no difference in the frequencies of major immune subsets between responders and non-responders, as analysed by the cluster abundance test in Citrus, with no specific model generated.

In the anti-PD-1 treated cohort, no specific predictive model was produced. This result is in accordance with the multivariate analysis that also did not yield any strong correlatives. In spite of its limitations, Citrus served as an unsupervised validation of the results obtained from manual gating of the CyTOF data.

### Effect of prior anti-CTLA-4 therapy on response to subsequent anti-PD-1 treatment

One patient from the anti-PD-1 group in this study initially received anti-CTLA-4, but developed severe immune-related colitis and had to discontinue therapy. The patient later developed additional metastasis and started anti-PD-1 (22 months after the end of anti-CTLA-4) therapy and had a partial response within 3 months. Both pre-anti-CTLA-4 and pre-anti-PD-1 PBMC from this patient were evaluated in this study. The two pre-treatment blood draws were 24 months apart and there was no recorded therapeutic intervention between the two therapies. Comparison of the viSNE map of the two samples revealed very low monocyte counts in the pre-anti-CTLA-4 PBMC, but not in the pre-anti-PD-1 sample (Additional file 1: Figure S11), which was confirmed by manual gating (data not shown). Also, we observed a significant reduction of CD14^−^CD33^+^ myeloid cells in the pre-anti-PD-1 sample versus pre-anti-CTLA-4 PBMC. This highlights the power of viSNE in identifying novel unanticipated cell populations. Whether these myeloid cells are functionally suppressive or not can only be determined after further characterization. Other changes observed in the pre-anti-PD-1 PBMC include an increase in CD56^hi^ NK cells, and increased CD27^+^ and CCR7^+^ CD4^+^ cells. We hypothesize that this altered immune signature, potentially a result of the anti-CTLA-4 treatment, played a role in the positive clinical outcome after the PD-1 therapy. Our data suggest that, while the anti-CTLA-4 treatment did not halt disease progression, it may have reshaped the immune system, and thereby allowed the patient to have a partial response (PR) to the subsequent anti-PD-1 treatment. However, we did not see general group-level differences between treatment naïve patients and those who had previously received anti-CTLA-4 (data not shown), indicating that there may be case-by-case variations in the process of immune re-shaping by prior immunotherapy.

## Discussion

In this study, we report one of the first CyTOF analyses on clinical samples from melanoma patients treated with checkpoint blockade. Using two cohorts of patients treated with anti-CTLA-4 or anti-PD-1 monotherapy, we discovered completely different biomarker candidates for the two immunotherapies. For anti-CTLA-4, we found differences in CD4^+^ and CD8^+^ memory T cells subsets between responders and non-responders. On the other hand, responders and non-responders to anti-PD-1 differed in CD69^+^ and MIP1β^+^ NK cell populations. Due to the high dimensionality afforded by CyTOF, this technology can enable us to detect novel (and often unexpected) cell populations that play a role in anti-tumor immunity. This, in turn will help in expediting clinical biomarker discovery, which is critical to the implementation of cancer immunotherapy.

One of the major challenges in dealing with CyTOF data, is performing high-dimensional data analysis [27]. viSNE offers visual presentation of the overall immune landscape but does not provide statistical analyses. Citrus is an automated algorithm designed to predict the difference between two datasets without bias from investigators. However, this algorithm has limitations, including those associated with the way that clusters are defined. In our analysis, viSNE was used for visualization, and Citrus was used for verification purposes only. Supervised manual gating and multivariate analysis, which were done by scientists with relevant expertise, were still the most trusted approach to identify parameters that correlate with clinical response. In this study, we discovered that a higher frequency of memory T cells in baseline PBMC is a potential biomarker candidate to predict clinical response to anti-CTLA-4 treatment in advanced melanoma patients. On the other hand, in anti-PD-1 treated patients, higher frequency of NK cells (CD69^+^ and MIP-1β ^+^) upon PMA + Ionomycin stimulation ex vivo, potentially correlates with positive response to therapy.

A recent study in our lab has shown that CD8^+^ Tcm cells were important in predicting response to anti-CTLA-4 and radiation combination therapy [28]. Another research study published recently also drew a correlation between CD45RO^+^CD8^+^ memory T cells and clinical response to anti-CTLA-4 therapy, but not anti-PD-1 therapy in melanoma patients [29]. In this study, we report that responders to anti-CTLA-4 monotherapy have high baseline frequencies of CD4^+^ and CD8^+^ T cell memory subsets, indicating that these memory subsets play an important role in anti-CTLA4 blockade, and warrant further investigation. Previous research in our lab has shown that the addition of bevacizumab, an angiogenesis blockade agent targeting Vascular Endothelial Growth Factor (VEGF), to anti-CTLA-4 therapy increased memory subsets in both the CD4^+^ and CD8^+^ T cell compartments [30]. The findings described in this study may provide an additional explanation for the observed synergy between checkpoint blockade and angiogenesis blockade. It will be interesting to determine if higher baseline memory T-cell subsets can be a consistent predictive biomarker for anti-CTLA-4 immunotherapy. In addition, recent reports suggested that anti-PD-1 antibody from different sources may have varying effects [31]. In light of the complexity of the mechanism of action of checkpoint blockade agents, the initial observations described here will require further confirmation in prospective cohorts.

Though T cells are believed to play the primary role in the anti-tumor immunity of checkpoint blockade therapies, the potential role of non-T cell populations cannot be excluded. We found that a higher frequency of CD69^+^ and MIP-1β^+^ NK cells upon in vitro stimulation is associated with better clinical response to anti-PD-1. CD69 is an activation marker for lymphocytes including NK cells. MIP-1β is a chemokine produced by macrophages, T and B cells, neutrophils, dendritic cells and NK cells. It recruits pro-inflammatory cells, and induces the production of pro-inflammatory cytokines [32]. It is also important for the transendothelial migration of NK cells, and also promotes Th1 skewing, which can aid anti-tumor immunity. It is reasonable to assume that the CD69^+^ and MIP-1β^+^ NK cells represent more functionally active NK populations. However, our multivariate analysis did not yield a strong predictive model for response to anti-PD-1 therapy using either PMA + Ionomycin stimulated data or unstimulated data.

In a recent anti-PD-1 biomarker study, high relative lymphocyte counts (≥17.5%) and high relative eosinophil counts (≥1.5%) were found to be predictors of better survival [19]. Another study by Martens et al. showed that low absolute monocyte count and high relative lymphocyte count were significantly associated with better survival in anti-CTLA-4 treated melanoma patients [18]. Our study did not reveal predictive value for lymphocyte and monocyte frequencies in the anti-CTLA-4 or anti-PD-1 cohorts (Additional file 1: Figure S2B and C). However, it should be noted that relative subset frequency or percentage was examined in this study, and is a different measurement from absolute cell count, which was the focus of the other studies. Also, our sample processing protocol included an overnight rest after thawing of samples. This could have potentially led to losses and variability in monocyte populations, making it hard to detect clear differences between responders and non-responders. Martens et al. also reported a correlation between Treg frequencies with overall survival of patients. However, our data did not show significant differences in Tregs between responders and non-responders (Additional file 1: Figure S7). One possible reason for this is that we defined Tregs as CD25^+^CD127^lo^ CD4^+^ T cells (our staining panel did not include FoxP3 which, being a transcription factor, requires harsher permeabilization conditions). Furthermore, we used a dichotomous stratification of patient outcome, whereas the other group performed a correlative analysis of these parameters with overall survival of patients. Finally, our assay platform is also different from these previous studies. Further investigation is needed to truly reveal all possible correlative parameters that could predict response to immunotherapy.

## Conclusions

Our findings suggest that response to anti-CTLA-4 and anti-PD-1 in advanced melanoma patients is complex and multifaceted. An improved understanding of factors that influence the ability to mount an effective anti-tumor response can inform our therapeutic strategies for melanoma and other diseases. Some of the findings in this study are partly validated by another study published recently which showed that memory subsets were predictive of response to CTLA-4 blockade in melanoma patients [28]. Further studies using larger patient cohorts and different types of cancer will also be invaluable in validating these candidate biomarkers, and are already underway in our laboratory. These long-term rigorous biomarker discovery efforts will not only help in patient selection, but will also provide deeper insight into the mechanism of action of these and other checkpoint blockade immunotherapies.

## Additional file

Additional file 1: **Figure S1.** Kaplan-Meier analysis of overall survival and progression-free survival in the anti-CTLA-4 and anti-PD-1 treated patient cohorts. **Figure S2.** Frequencies of major immune cell subsets in responders and non-responders to anti-CTLA-4 or anti-PD-1 therapy. **Figure S3.** viSNE analysis of CD45RA expression on CD4^+^ and CD8^+^ T cells in patients treated with anti-CTLA-4. **Figure S4.** Frequencies of memory/non-memory T cells in melanoma patients treated with anti-PD-1. **Figure S5.** CD69^+^MIP-1β^+^ NK cells are a distinct population. **Figure S6.** CD69 and MIP-1β expressing NK cells in melanoma patients treated with anti-CTLA-4. **Figure S7.** Frequencies of regulatory T cells in baseline PMBC from anti-CTLA-4 and anti-PD-1 treated melanoma patients. **Figure S8.** Baseline CTLA-4 and PD-1 expression on T cells in anti-CTLA-4 and anti-PD-1 treated patients. **Figure S9.** PD-L1 and PD-L2 expression in monocytes and NK cells in anti-CTLA-4 and anti-PD-1 treated melanoma patients. **Figure S10.** Expression of Granzyme B in NK cells and CD8^+^ T cells in anti-CTLA-4 and anti-PD-1 treated patients. **Figure S11.** A comparison of viSNE maps between pre-anti-CTLA-4 PBMC and pre-anti-PD-1 PBMC from the same patient. (DOCX 3521 kb)
