# Supplementary material for: Distinct predictive biomarker candidates for response to anti-CTLA-4 and anti-PD-1 immunotherapy in melanoma patients
Source: J Immunother Cancer. 2018 Mar 6;6:18. doi: 10.1186/s40425-018-0328-8 (PMC5840795; doi:10.1186/s40425-018-0328-8)
Supplement: Supplementary file 1 — Figure S1. Kaplan-Meier analysis of overall survival and progression-free survival in the anti-CTLA-4 and anti-PD-1 treated patient cohorts. Figure S2. Frequencies of major immune cell subsets in responders and non-responders to anti-CTLA-4 or anti-PD-1 therapy. Figure S3. viSNE analysis of CD45RA expression on CD4+ and CD8+ T cells in patients treated with anti-CTLA-4. Figure S4. Frequencies of memory/non-memory T cells in melanoma patients treated with anti-PD-1. Figure S5. CD69+MIP-1β+ NK cells are a distinct population. Figure S6. CD69 and MIP-1β expressing NK cells in melanoma patients treated with anti-CTLA-4. Figure S7. Frequencies of regulatory T cells in baseline PMBC from anti-CTLA-4 and anti-PD-1 treated melanoma patients. Figure S8. Baseline CTLA-4 and PD-1 expression on T cells in anti-CTLA-4 and anti-PD-1 treated patients. Figure S9. PD-L1 and PD-L2 expression in monocytes and NK cells in anti-CTLA-4 and anti-PD-1 treated melanoma patients. Figure S10. Expression of Granzyme B in NK cells and CD8+ T cells in anti-CTLA-4 and anti-PD-1 treated patients. Figure S11. A comparison of viSNE maps between pre-anti-CTLA-4 PBMC and pre-anti-PD-1 PBMC from the same patient. (DOCX 3521 kb) [file 40425_2018_328_MOESM1_ESM.docx]

Additional file 1 for

**Distinct predictive biomarker candidates for response to anti-CTLA-4 and anti-PD-1 immunotherapy in melanoma patients**

**Running Title:** CyTOF predictive biomarkers for melanoma checkpoint blockade

Priyanka B. Subrahmanyam*^1^, Zhiwan Dong*^2^, Daniel Gusenleitner^2^, Anita Giobbie-Hurder^2,3^, Mariano Severgnini^2^, Jun Zhou^4^, Michael Manos^2^, Lauren M. Eastman^2^, Holden T. Maecker^1^, F. Stephen Hodi^‡2,4,5^

*These authors contributed equally

^‡^Corresponding author: F. Stephen Hodi

Email: [stephen_hodi@dfci.harvard.edu](mailto:stephen_hodi@dfci.harvard.edu)

^1^Institute for Immunity, Transplantation, and Infection, Stanford University School of Medicine, Stanford, California.

^2^Center for Immuno-oncology, Dana-Farber Cancer Institute and Harvard Medical School, Boston, Massachusetts.

^3^Department of Biostatistics and Computational Biology, Dana-Farber Cancer Institute, Boston, Massachusetts.

^4^Department of Medical Oncology, Dana-Farber Cancer Institute and Harvard Medical School, Boston, Massachusetts.

^5^Melanoma Disease Center, Dana-Farber Cancer Institute and Harvard Medical School, Boston, Massachusetts.

This file includes:

Fig. S1. Kaplan-Meier analysis of overall survival and progression-free survival in the anti-CTLA-4 and anti-PD-1 treated patient cohorts.

Fig. S2. Frequencies of major immune cell subsets in responders and non-responders to anti-CTLA-4 or anti-PD-1 therapy.

Fig. S3. viSNE analysis of CD45RA expression on CD4^+^ and CD8^+^ T cells in patients

treated with anti-CTLA-4.

Fig. S4. Frequencies of memory/non-memory T cells in melanoma patients treated with anti-PD-1.

Fig. S5. CD69^+^MIP-1β^+^ NK cells are a distinct population.

Fig. S6. CD69 and MIP-1β expressing NK cells in melanoma patients treated with anti-CTLA-4.

Fig. S7. Frequencies of regulatory T cells in baseline PMBC from anti-CTLA-4 and anti-PD-1 treated melanoma patients.

Fig. S8. Baseline CTLA-4 and PD-1 expression on T cells in anti-CTLA-4 and anti-PD-1 treated patients.

Fig. S9. PD-L1 and PD-L2 expression in monocytes and NK cells in anti-CTLA-4 and anti-PD-1 treated melanoma patients.

Fig. S10. Expression of Granzyme B in NK cells and CD8^+^ T cells in anti-CTLA-4 and anti-PD-1 treated patients.

Fig. S11. A comparison of viSNE maps between pre-anti-CTLA-4 PBMC and pre-anti-PD-1 PBMC from the same patient.

**Figure S1.** Kaplan-Meier analysis of overall survival and progression-free survival in anti-CTLA-4 and anti-PD-1 treated cohorts. **A**, Median overall survival of patients in the anti-CTLA-4 treated cohort was 46.7 months. **B**, Median overall survival of patients in the anti-PD-1 treated cohort was not yet reached at 42 months. **C**, Median progression-free survival of patients in the anti-CTLA-4-treated cohort was 5 months. **D**, Median progression-free survival of patients in the anti-PD-1-treated cohort was 7 months.

**Fig. S1**


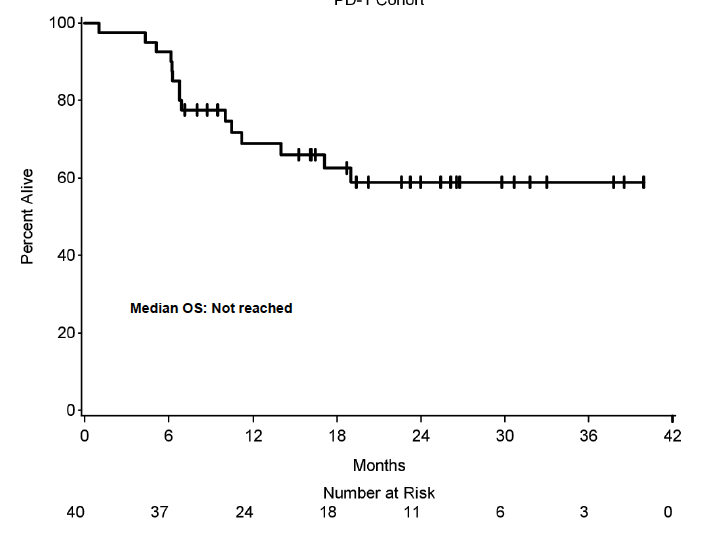

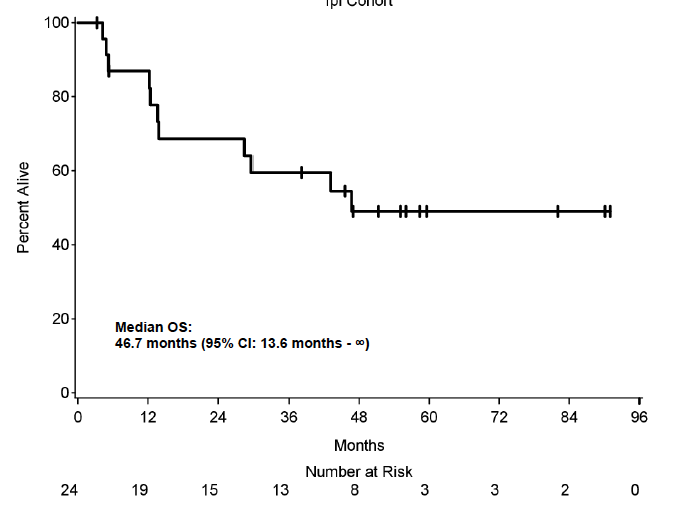


Anti-PD-1 cohort

Overall Survival


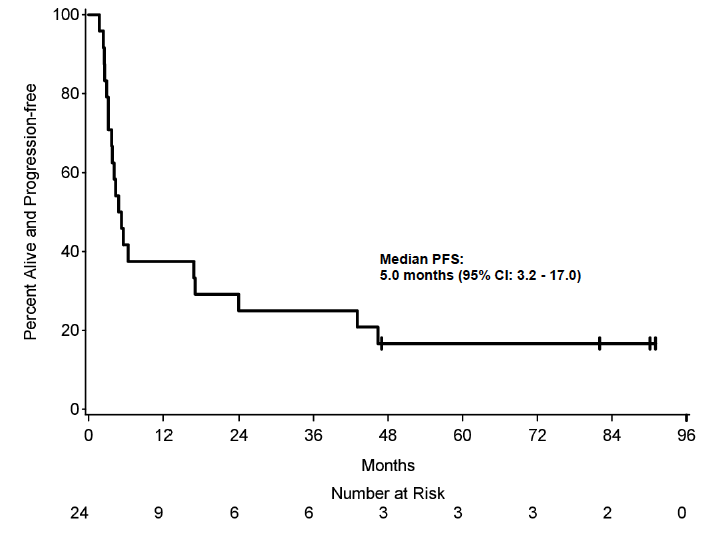


Anti-CTLA-4 cohort

Progression-free Survival

Anti-CTLA-4 cohort

Overall Survival


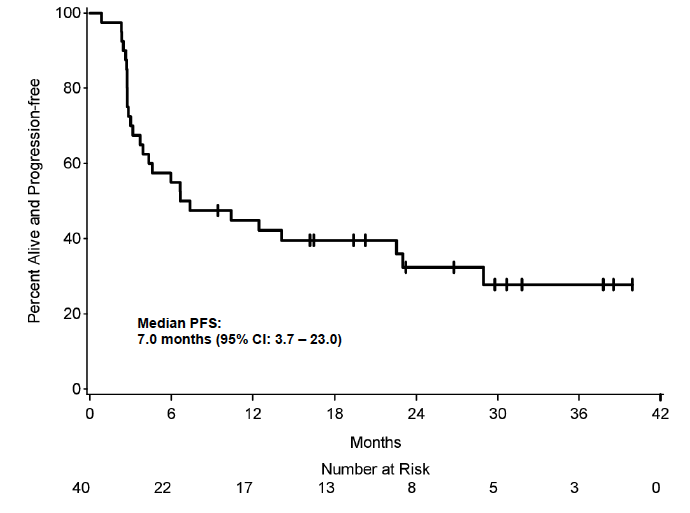


Anti-PD-1 cohort

Progression-free Survival

**A**

**B**

**C**

**D**

**Figure S2.** Frequencies of major immune cell subsets in responders and non-responders to anti-CTLA-4 or anti-PD-1 therapy. CyTOF data were analyzed using FlowJo and the frequencies of major immune cell subsets are shown. **A,** Live intact singlets gated as shown in Fig. 1A, were further gated on CD14 and CD33. *a)* CD14^+^CD33^+^ were classified as monocytes while other cells were classified as lymphocytes. *b）*CD3 expression on lymphocytes was used to identify CD3^+^ T cell and CD3^-^ non-T cell populations. *c）*CD4 and CD8 expression on T cells was used to identify CD4^+^ and CD8^+^ T cell populations. Frequencies of **B,** lymphocytes; and **C,** monocytes as gated in *a)*. **D,** Frequencies of B cells gated as CD19^+^CD20^+^ from CD3^-^ lymphocytes are shown as a scatter plot; **E,** Natural Killer (NK) cells were gated as CD56^+^CD16^+^ from CD3^-^ lymphocytes, and frequencies are shown; **F,** Frequencies of natural killer T (NKT) cells gated as CD56^+^ T cells; **G,** Frequencies of γδ T cells gated as TCRγδ^+^ T cells are shown.

**Fig. S2**

**Figure S3.** viSNE analysis of CD45RA expression on CD4^+^ and CD8^+^ T cells in patients treated with anti-CTLA-4. CD4^+^ and CD8^+^ T cell subsets were selected for viSNE map generation. Coloring was based on the 153Eu CD45RA channel to demonstrate CD45RA expression. The expression level, shown as 153Eu intensity (red) can be compared between responders and non-responders to anti-CTLA-4 therapy.

**Fig. S3**

**Figure S4.** Frequencies of memory/non-memory T cells in melanoma patients treated with anti-PD-1. FlowJo analysis of CyTOF data from baseline PBMC of melanoma patients treated with anti-PD-1. Frequencies of **A,** CD45RA^+^ cells in CD4^+^ and CD8^+^ T cell compartments; **B,** CD45RA^-^ cells in CD4^+^ and CD8^+^ T cell compartments. **(C-F)** Memory subsets of CD4^+^ and CD8^+^ T cells in responders *vs.* non-responders to anti-PD-1. **C,** Frequencies of naïve (CD45RA^+^CCR7^+^) and Central Memory (Tcm, CD45RA^-^CCR7^+^) CD4^+^ T cells. **D,** Frequencies of Effector Memory (Tem, CD45RA^-^CCR7^-^) and Terminal Effector (Teff, CD45RA^+^CCR7^-^) CD4^+^ T cells. **C,** Frequencies of naïve (CD45RA^+^CCR7^+^) and Central Memory (Tcm, CD45RA^-^CCR7^+^) CD8^+^ T cells. **D,** Frequencies of Effector Memory (Tem, CD45RA^-^CCR7^-^) and Terminal Effector (Teff, CD45RA^+^CCR7^-^) CD8^+^ T cells.

**Fig. S4**

**Figure S5.** CD69^+^MIP-1β^+^ NK cells are a distinct population. Representative examples of FlowJo plots showing expression of CD69 and MIP-1β in NK cells following PMA/Ionomycin stimulation. **A,** Expression of MIP-1β (left) and CD69 (right) in a non-responder (top row) and a responder (bottom row) to anti-PD-1 treatment. **B,** Histogram comparison of MIP-1β (left) and CD69 (right) expression in a non-responder (grey area with dotted line) and a responder (white area with solid line) to anti-PD-1 treatment. **C**, Dot plots for the CD69^+^MIP-1β^+^ NK cell subset in a non-responder (top), a responder (middle) and a healthy donor (bottom).

**Fig. S5**

**Figure S6.** CD69 and MIP-1β expressing NK cells in melanoma patients treated with anti-CTLA-4. Pre-treatment PBMC from melanoma patients who were responders or non-responders to anti-CTLA-4 therapy were stimulated with PMA+Ionomycin *ex vivo*, and analyzed by CyTOF. Frequencies of **A,** CD69^+^, **B**, MIP-1β^+^, and **C,** CD69^+^MIP-1β^+^ NK cells are shown.

**Fig. S6**

**Figure S7.** Frequencies of regulatory T cells in baseline PMBC from anti-CTLA-4 and anti-PD-1 treated melanoma patients. **A**, A representative dot plot showing the gating of regulatory T cells from total T cells: CD4^+^CD25^+^CD127^lo^ **B,** Frequencies of regulatory CD4^+^ T cells in responders and non-responders to anti-CTLA-4 or anti-PD-1 immunotherapy.

**Fig. S7**

**Figure S8**. Baseline CTLA-4 and PD-1 expression on T cells in anti-CTLA-4 and anti-PD-1 treated patients. Frequencies of **A,** CTLA-4^+^ CD4^+^ T cells; **B,** CTLA-4^+^ CD8^+^ T cells; **C,** PD-1^+^ CD4^+^ T cells and **D,** PD-1^+^ CD8^+^ T cells in baseline PBMC from patients treated with anti-CTLA-4 or anti-PD-1 immunotherapy. Patient groups are divided into responders and non-responders for each therapy.

**Fig. S8**

**Figure S9.** PD-L1 and PD-L2 expression in monocytes and NK cells in anti-CTLA-4 and anti-PD-1 treated melanoma patients. Frequencies of **A**, PD-L1^+^ and **B,** PD-L2^+^ monocytes in baseline PBMC from patients who were responders or non-responders to anti-CTLA-4 or anti-PD-1 therapy. **C**, PD-L1^+^ or **D,** PD-L2^+^ NK cells in baseline PBMC from patients treated with anti-CTLA-4 or anti-PD-1.

**Fig. S9**

**Figure S10.** Expression of Granzyme B in NK cells and CD8^+^ T cells in anti-CTLA-4 and anti-PD-1 treated patients. **A,** Frequencies of Granzyme B^+^ NK cells and **B,** Frequencies of Granzyme B^+^CD8^+^ T cells in baseline PBMC from melanoma patients treated with anti-CTLA-4 or anti-PD-1 immunotherapy. **p<0.01

**Fig. S10**

**Figure S11.** A comparison of viSNE maps between pre-anti-CTLA-4 PBMC and pre-anti-PD-1 PBMC from the same patient. A patient dropped out of the anti-CTLA-4 monotherapy due to immune-related adverse events, and was treated with anti-PD-1 therapy 22 months after the end of anti-CTLA-4 treatment. The pre-anti-PD-1 blood was drawn 24 months after the pre- anti-CTLA-4 blood draw. No other therapeutic intervention was recorded between the two therapies. **A,** viSNE maps of PBMC from pre-anti-CTLA-4 (left) and pre-anti-PD-1 (right). **B,** Major immune subsets have been outlined. Each dot represents an event (cell).

**Fig. S11**
